# Low‐Noise Dual‐Band Polarimetric Image Sensor Based on 1D Bi_2_S_3_ Nanowire

**DOI:** 10.1002/advs.202100075

**Published:** 2021-05-21

**Authors:** Wen Yang, Juehan Yang, Kai Zhao, Qiang Gao, Liyuan Liu, Ziqi Zhou, Shijun Hou, Xiaoting Wang, Guozhen Shen, Xinchang Pang, Qun Xu, Zhongming Wei

**Affiliations:** ^1^ School of Materials Science and Engineering Zhengzhou University Zhengzhou 450052 China; ^2^ State Key Laboratory of Superlattices and Microstructures Institute of Semiconductors Chinese Academy of Sciences Beijing 100083 China; ^3^ Center of Materials Science and Optoelectronics Engineering University of Chinese Academy of Sciences Beijing 100049 China; ^4^ Henan Institute of Advanced Technology Zhengzhou University Zhengzhou 450052 China

**Keywords:** Bi_2_S_3_, image sensors, low‐frequency noise, nanowires, polarization‐sensitive

## Abstract

With the increasing demand for detection accuracy and sensitivity, dual‐band polarimetric image sensor has attracted considerable attention due to better object recognition by processing signals from diverse wavebands. However, the widespread use of polarimetric sensors is still limited by high noise, narrow photoresponse range, and low linearly dichroic ratio. Recently, the low‐dimensional materials with intrinsic in‐plane anisotropy structure exhibit the great potential to realize direct polarized photodetection. Here, strong anisotropy of 1D layered bismuth sulfide (Bi_2_S_3_) is demonstrated experimentally and theoretically. The Bi_2_S_3_ photodetector exhibits excellent device performance, which enables high photoresponsivity (32 A W^−1^), *I*_on_/*I*_off_ ratio (1.08 × 10^4^), robust linearly dichroic ratio (1.9), and Hooge parameter (2.0 × 10^−5^ at 1 Hz) which refer to lower noise than most reported low‐dimensional materials‐based devices. Impressively, such Bi_2_S_3_ nanowire exhibits a good broadband photoresponse, ranging from ultraviolet (360 nm) to short‐wave infrared (1064 nm). Direct polarimetric imaging is implemented at the wavelengths of 532 and 808 nm. With these remarkable features, the 1D Bi_2_S_3_ nanowires show great potential for direct dual‐band polarimetric image sensors without using any external optical polarizer.

## Graphical abstract

A low noise of the 1D bismuth sulfide (Bi_2_S_3_) nanowire image sensor ensures direct polarimetric imaging with high contrast and distinct targets. Direct polarimetric imaging is implemented at the wavelengths of 532 and 808 nm, respectively. Given these remarkable photodetector features, the low‐dimensional Bi_2_S_3_ nanowires will hold great promise for dual‐band direct polarimetric imaging application.

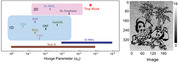

## 1 Introduction

Dual‐band polarimetric image sensor can process signals from different wavelengths to better identify targets, which is of great significance for the development of astronomy, biological diagnosis, land cover classification, and 3D surface reconstruction.^[^ ^1^, ^2^, ^3^, ^4^, ^5^, ^6^ ^]^ Dual‐band image sensor is currently dominated by mature material technologies such as HgCdTe^[^ ^7^ ^]^ and type‐II superlattices.^[^ ^8^ ^]^ However, these kind of image sensors suffer from low production yield, high fabrication complexity, and high cost, and so their widespread application has been limited. Emerging low‐dimensional materials provide another route to meet these demands, due to their native in‐plane anisotropy crystal structure for high polarization sensitivity, the ease of integrating on the silicon platform for good complementary metal‐oxide‐semiconductor compatibility, and strong quantum confinement for excellent photoelectric performances. Recently, some low‐dimensional materials with intrinsic in‐plane anisotropy structure exhibit great potential to realize direct polarized photodetection without the use of polarization filters.^[^ ^9^, ^10^, ^11^, ^12^, ^13^, ^14^, ^15^, ^16^ ^]^ Nevertheless, there are three main hurdles for polarimetric image sensors to achieve high‐resolution imaging at multispectral. First, the controllable growth of large‐size low‐dimensional single crystals is still a challenge. Second, to realize the different wavebands polarimetric imaging, the sensor required high photoresponsivity and robust polarization sensitivity in the broadband spectrum. Third, the large dark current in sensor can provide high noise (and consequently, the high signal‐to‐noise ratio). The level of noise is closely related to accuracy and sensitivity of image sensor.^[^ ^17^, ^18^ ^]^ Thus, exploring materials with intrinsic in‐plane anisotropy structure for multispectral polarimetric image sensor with low noise is a great challenge.

To address these three hurdles and facilitate the development of polarimetric image sensors, 1D layered nanomaterial with an in‐plane anisotropy structure is proposed for dual‐band polarimetric image sensors. First, 1D nanomaterials usually have a large surface‐to‐volume ratio and strong light–matter interaction, which can provide high photoresponsivity.^[^ ^19^ ^]^ Second, the lack of dangling bonds on the surface of the 1D layered nanomaterials can effectively lower 1/*f* noise from generation‐recombination, reducing surface‐induced performance degradation, facilitating easy integration with other substrates or materials.^[^ ^20^, ^21^, ^22^ ^]^ Third, comprehensive spectral, spatial, and polarization information effectively improves the contrast between target and background.

As an important member of Group VA–VIA semiconductor, bismuth sulfide (Bi_2_S_3_) has drawn tremendous interest as its competitive features, such as narrow‐bandgap range (1.3–1.7 eV), strong in‐plane anisotropy, high absorption coefficient (≈10^5^ cm^–1^), high carrier mobility (≈200 cm^2^ V^−1^ s^−1^), and a wide‐band spectral response. In addition, the advantages of nontoxicity, high crust abundance, as well as the facile synthetic route make 1D Bi_2_S_3_ nanowire (NW) an ideal candidate for exploring environment‐friendly, low‐cost, and high‐efficiency image sensors.^[^ ^23^, ^24^, ^25^, ^26^, ^27^ ^]^ Furthermore, sulfurization can reduce trap density of states of the Bi_2_S_3_ and lower 1/*f* noise.^[^ ^28^ ^]^

In this work, 1D Bi_2_S_3_ NWs with high quality were successfully synthesized via a sulfur‐assisted vapor transport growth method. The results of polarization‐resolved optical microscopy (PROM) and angle‐resolved polarized Raman spectroscopy (ARPRS) show that Bi_2_S_3_ NWs have significant in‐plane anisotropy. Significantly, due to reduced defects of 1D Bi_2_S_3_ NW via a facile sulfurization approach, the image sensor shows a superior device performance in terms of high photoresponsivity, broadband, high dichroic ratio, and excellent polarization imaging in visible or short‐wave infrared. Moreover, the image sensor exhibits low noise, which is better than those of most reported low‐dimensional materials and comparable to the ultralow noise of silicon nanowires. Our work paves a potential pathway toward constructing high‐resolution dual‐band polarimetric image sensors based on 1D materials.

## 2 Results and Discussion

**Figure 1:**
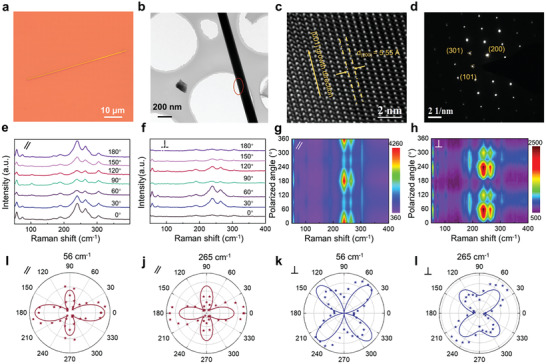
Characterization of Bi_2_S_3_ crystal. a) Typical microscopy optical image of single Bi_2_S_3_ Nanowire. b) Low‐magnification transmission electron microscopy (TEM) of the Bi_2_S_3_ NW. c) High‐resolution transmission electron microscopy (HTEM) image of the Bi_2_S_3_ NW. d) SAED pattern of the Bi_2_S_3_ NW. Angle‐resolved Raman spectra and the corresponding contour map of the Bi_2_S_3_ NW measured in the e,g) parallel and f,h) cross‐polarization configurations. i–l) Polar plot of the extracted measured and fitted peak intensities of the A_g_ (56 cm^−1^) and B_1g_ (265 cm^−1^) mode under the polarization configurations (parallel or cross). The stars were measured without background correction, and solid curves were fitted by the equation in Table S1 (Supporting Information).

To reduce sulfur defects in the as‐grown products, Bi_2_S_3_ NWs were directly grown on SiO_2_/Si substrate by sulfur‐assisted vapor transport method (Figure S1a, Supporting Information). The optical microscopy image (**Figure** **1**a) and atomic force microscopy image (Figure S1b, Supporting Information) show the Bi_2_S_3_ NW with a length of about 60 µm and a diameter of 123 nm. In addition, from X‐ray diffraction (XRD) (Figure S2, Supporting Information) analysis, all the diffraction peaks agree with the standard orthorhombic Bi_2_S_3_ pattern (JCPDS 17‐0320, cell constants *a* = 11.149, *b* = 11.304, and *c* = 3.981) and without secondary phases, suggesting the excellent crystallinity and phase purity of the as‐grown Bi_2_S_3_ NWs.

Transmission electron microscopy (TEM) equipped with energy‐dispersive X‐ray (EDX) spectroscopy was performed to further characterize the crystal microstructure and growth orientation at the atomic level. Figure 1b exhibits the low‐magnification TEM image of a typical Bi_2_S_3_ NW. The high‐resolution transmission electron microscopy (HRTEM) image (Figure 1c) obtained from the marked ellipse region in Figure 1b clearly shows distinct and perfect lattice fringes over large lateral scale. The measured lattice spacing of 5.55 Å agrees with the (220) plane of the orthorhombic Bi_2_S_3_. Moreover, the corresponding selected area electron diffraction (SAED) pattern in Figure 1d presents only a set of clear and sharp diffraction spots, which are well indexed to (101), (301), and (200) plane, and square symmetry. Moreover, high‐angle annular dark‐field (HAADF) TEM images with the corresponding elemental maps are shown in Figure S3a–d (Supporting Information). Bi and S elements are uniformly distributed over all Bi_2_S_3_ NW. The EDS reveals obvious peaks of Bi and S with a Bi:S chemical composition of about 2:3 (Figure S3e, Supporting Information). Together, taking all these results suggests the as‐grown 1D Bi_2_S_3_ has high quality, good uniformity, and good chemical stoichiometry. The Raman scattering spectrum also evaluated the structure of the as‐grown Bi_2_S_3_ NWs. Theoretical analysis indicates that there are 30 Raman active vibrational modes for a Bi_2_S_3_ cell with 20 atoms, consisting of ten A_g_ modes, ten B_1g_ modes, five B_2g_ modes, and five B_3g_ modes.^[^ ^29^ ^]^ The traditional Raman spectrum (Figure S3f, Supporting Information) under laser (785 nm) shows distinct Raman peaks at 56, 73, 102, 188, 240, and 265 cm^−1^ are all consistent with the previous studies. ^[^ ^25^, ^30^ ^]^ In addition, X‐ray photoelectron spectroscopic (XPS) measurement is further performed to reveal the compositional and chemical state of the product. Figure S4 (Supporting Information) presents a typical XPS pattern of the as‐prepared product and is consistent with previously observed in Bi_2_S_3_. ^[^ ^28^ ^]^

Low symmetry of the Bi_2_S_3_ crystal structure indicates that its phonon vibration should be anisotropic. Anisotropic optical properties of Bi_2_S_3_ were characterized by angular‐resolved polarized Raman spectroscopy (Figure 1e,f) under parallel and cross‐polarization configurations. In these measurements, the incident laser was polarized along the horizontal orientation and a linear polarizer was placed in front of the Raman detector, which enables us to record the scattered Raman signals that polarized parallel and perpendicular to the incident laser polarization (denote as parallel and cross‐polarization configurations, respectively). Figure 1g,h shows the contour map of polarized Raman intensities at different rotation angles under parallel and cross‐polarization configurations, respectively. It is clear that the peak intensities of A_g_ (56 cm^−1^) and B_1g_ (265 cm^−1^) vary periodically with the polarized angle of the incident light.

According to the classical Placzek model, the Raman scattering intensity can be expressed as *I*∝|*e*_i_ · *R* · *e*_s_|^2^, where *e*_i_ and *e*_s_ are the electric polarization vectors unit vectors of the incident and scattered light,^[^ ^10^, ^31^ ^]^ respectively, and the *R* is the second‐order Raman tensor of the corresponding Raman vibration mode. When defining *θ* as the angle between incident polarization and *c*‐axis, the incident light polarization can be described as *e*_i_ = (cos *θ*, sin *θ*, 0), while the scattered light polarization can be denoted as *e*_s_ = (cos *θ*, sin *θ*, 0) or *e*_s_ = (−sin *θ*, cos *θ*, 0), under parallel or cross‐configurations. The Raman tensors of the A_g_ (56 cm^−1^) and B_1g_ (265 cm^−1^) photovibration modes of Bi_2_S_3_ can be denoted as follows

(1) $$R\left(A_{g}\right)=\left[\begin{array}{ccc} \left|a\right|e^{i\varphi_{a}} & 0 & 0\\ 0 & \left|b\right|e^{i\varphi_{b}} & 0\\ 0 & 0 & \left|c\right|e^{i\varphi } \end{array}\right]$$

(2) $$R\left(B_{1g}\right)=\left[\begin{array}{ccc} 0 & \left|d\right|e^{i\varphi_{d}} & 0\\ \left|d\right|e^{i\varphi_{d}} & 0 & 0\\ 0 & 0 & 0 \end{array}\right]\;$$

where *φ*_a_ *, φ*_b_ *, φ*_c_, and *φ*_d_ are the phase of the *a, b, c*, and *d*,^[^ ^32^ ^]^ respectively. Then, the Raman scattering intensities can be expressed by the following equations for A_g_ mode

(3) $$I\;\left(A_{g},∥\right)\propto {\left(\left|b\right|\sin^{2}\theta +\left|c\right|\cos\varphi_{\mathrm{cb}}\cos^{2}\theta \right)}^{2}+{\left|c\right|}^{2}\sin^{2}\varphi_{\mathrm{cb}}\cos^{4}\theta$$

(4) $$\left(A_{g},⊥\right)\propto \left[{\left(\left|b\right|-\left|c\right|\cos\varphi_{\mathrm{cb}}\right)}^{2}+{\left|c\right|}^{2}\sin^{2}\varphi_{\mathrm{cb}}\right]\sin^{2}\theta \cos^{2}\theta$$

and for *B*_1g_ mode

(5) $$I\;\left(B_{1g},∥\right)\propto d^{2}\sin^{2}2\theta$$

(6) $$I\;\left(B_{1g},⊥\right)\propto d^{2}\sin^{2}2\theta$$

Figure 1i–l shows the experimental values (star) and fitting lines of the Raman intensities varying with the polarized angle of the incident light in the polar coordinates. The experimental values and fitting lines possess the same variation trends.

Besides the anisotropic Raman property, anisotropic optical refraction of the Bi_2_S_3_ was further investigated via polarization‐resolved optical microscopy (PROM) measurement. The PROM can directly visualize optical anisotropy through the optical contrast due to the birefringence of anisotropic 2D materials. Figure S5 (Supporting Information) and Movie S1 (Supporting Information) show the transmitted PROM images of Bi_2_S_3_ NW at different sample rotation angles in steps of 10° in the cross mode. The refraction changes from the weakest to the strongest when the NW rotates 45° clockwise from parallel direction. All these data exhibit that the Bi_2_S_3_ NW is anisotropic in optical refraction. The anisotropic phonon vibration and anisotropic optical reflection provide us great opportunities to further explore anisotropy in a theoretical level.

**Figure 2:**
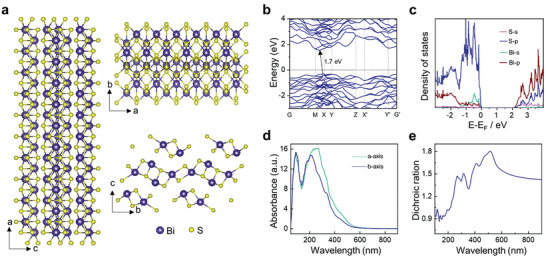
Theoretical calculations of bulk Bi_2_S_3_. a) Atomic structure of the orthorhombic Bi_2_S_3_ crystal. b) Band structure of layered Bi_2_S_3_. c) Calculated density of states (DOS). d) Absorbance along *a*‐axis and *b*‐axis for Bi_2_S_3_ in theory. e) The dichroic ration *α_a_*/*α_b_* extracted from Bi_2_S_3_ absorbance.

The band structure and optical properties of Bi_2_S_3_ were simulated via using the Vienna Ab initio Simulation Package (VASP) based on the density functional theory (DFT).^[^ ^33^, ^34^ ^]^ A schematic crystal structure in **Figure** **2**a shows two infinite ribbons (Bi_4_S_6_)*_n_* stack along the *a*‐axis through strong covalent Bi—S bond and hold together by Van der Waals forces in the *c*‐axis.^[^ ^35^ ^]^ The lattice parameters of the theoretical calculation model are *a* = 4.023 Å, *b* = 11.191 Å, and *c* = 11.733 Å. Figure 2b shows that Bi_2_S_3_ is a semiconductor with an indirect bandgap of 1.7 eV. As shown in Figure 2c, the electronic density of the state (DOS) of the Bi_2_S_3_ shows that the conduction band minimum (CBM) and valence band maximum (VBM) are mainly contributed by the p orbital states of Bi atoms and p orbital states of S atoms, respectively. Due to the structural asymmetry along the *a*‐axis and *b*‐axis, the anisotropic optical properties of Bi_2_S_3_ can be expected. The optical absorption coefficient of Bi_2_S_3_ is shown in Figure 2d by using the following formula

(7) $$\alpha \;\left(\omega \right)=\sqrt{2}\;\omega {\left[\sqrt{{\varepsilon_{1}}^{2}\left(\omega \right)+{\varepsilon_{2}}^{2}\left(\omega \right)}-\varepsilon_{1}\left(\omega \right)\right]}^{\frac{1}{2}}$$

where *ε*_1_(*ω*) and *ε*_2_(*ω*) represent the real parts and imaginary parts of the complex dielectric function, respectively. The result in Figure 2d indicates a distinct optical absorption coefficient (*α*) along the *a*‐axis and *b*‐axis. As shown in Figure 2e, the calculated dichroism ratio can reach the value of ≈1.75 and ≈1.6 with a wavelength at 532 and 808 nm, respectively. According to Fermi's Golden Rule, the *ac*‐plane and *bc*‐plane might have different optical properties to the external in‐plane polarization, leading to the linear dichroism of the Bi_2_S_3_. The corresponding formula is expressed as follows

(8) $$R\;=\left(\frac{2\pi }{\hbar }\right)\;\sum {\left|c\langle \left|H_{\mathrm{eR}}\right|\rangle \nu \right|}^{2}\delta \left(E_{c}\left(k_{c}\right)-E_{c}\left(k\right)-\hbar \omega \right)$$

Optical absorbance is a positive correlation with the parameter *R*.^[^ ^36^ ^]^ The distribution of charge density distribution along the *a*‐axis and *b*‐axis has different 〈*c*|*H*_eR_|*ν*〉, resulting from the different values of *R* for photon absorption per unit time in the *a*‐axis and *b*‐axis. The special bandgap and distinct optical absorption anisotropy in Bi_2_S_3_ prompt us to further explore in‐plane photoelectric properties along with different directions.

**Figure 3:**
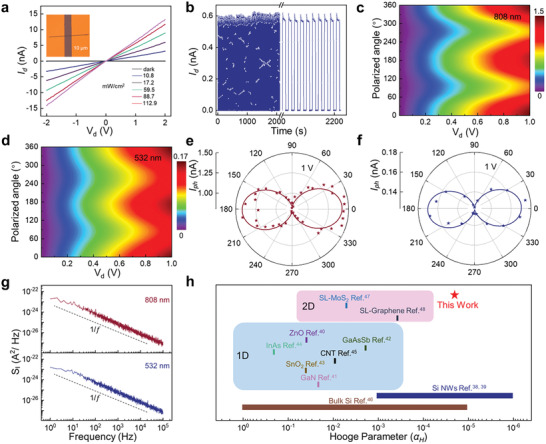
Optoelectronic characteristics and noise characteristics of Bi_2_S_3_ NW. a) *I*_d_ *–V*_d_ curves of Bi_2_S_3_ photodetector under 808 nm light illumination at different power densities. Inset is an OM image of the device. b) The stability of the Bi_2_S_3_ photodetector using an incident power density of 11.72 mW cm^−2^. c,d) 2D colormap of the anisotropic photocurrent of Bi_2_S_3_ NWs under the 808 and 532 nm at bias from 0 to1 V, respectively. e,f) Polarization‐sensitive photocurrent of the same sample measured from 0° to 360° at *V*_d_ = 1 V. Polar plot of polarization‐dependent photocurrent under 808 and 532 nm, respectively. The line is the fitting results using the sinusoidal function. g) Noise power density as a function of Hertz at 0.5 *V*_d_ under 808 and 532 nm, respectively. h) Comparison of the noise magnitude in terms of Hooge parameter *α*_H_ for various 1D, 2D semiconductors and Si.

To investigate the photoelectric properties of the Bi_2_S_3_ NWs, we fabricated a two‐terminal device on SiO_2_/Si substrate by the lithography and metal electrode evaporation process. Figure S6a (Supporting Information) shows the testing setup of photoresponse as a function of the incident light with different polarization angles. Linearly polarized light is obtained by passing a laser through a polarizer (Glan‐Taylor prism) and a half‐wave plate (HWP). The Glan‐Taylor prism's extinction ratio reaches 10^5^:1, ensuring that the emitted light through the Glan‐Taylor prism is linearly polarized. The polarized angle of linearly polarized light is changed by rotating the HWP, which does not change the intensity of linearly polarized light. **Figure** **3**a presents the *I–V* characteristics of the device under dark and light irradiation with power density range from 10.08 to 112.9 mW cm^−2^ under 808 nm light illumination. The time‐resolved photoresponse of the Bi_2_S_3_ photodetector was measured under 808 nm laser by periodically turning it on and off. The repeatable response with different *V*_d_ (0.25, 0.5, 1 V) under 59.5 mW cm^−2^ illumination (Figure S6b, Supporting Information) and the reproducible current in each switching cycle could be observed during 220 cycles (Figure 3b), suggesting good repeatability and reproducibility. Importantly, there is little deviation after 220 cycles, revealing the high stability of the photodetector. The photoresponse of device is strongly dependent on the power intensity of the incident light, as shown in Figure S6c (Supporting Information). The photocurrent (*I*_ph_) as a function of the incident light intensity (*P*) can be described by fitting it with the following equation, *I*_ph_ = *αP* ^*β*^ where *β* is the index of the power and *α* is a constant. The calculated value of *β* is 0.6. Besides the trap states between the substrate and Bi_2_S_3_, the nonunity exponent may also be related to the defects that induce charge trapping; similar sublinear responses have also been observed in other low dimensional materials. ^[^ ^23^, ^24^, ^37^ ^]^ The photoelectric properties of the photodetector (Figure S7a–d, Supporting Information) were also measured under 532 nm laser light irradiation, exhibiting an excellent photoswitching ratio of 1.08 × 10^4^ and high photoresponsivity of 32 A W^−1^. The response speed can be evaluated by analyzing the rising and falling edges of an individual response cycle, which are defined as the time intervals for the response to rise 10% to 90% and decay from 90% to 10% of its peak value. According to the above definition, a rise/fall time of about 20/10 ms is achieved (Figure S8, Supporting Information). Responsivity (*R*) is a critical parameter to evaluate the performance of photodetector, which is calculated by the following formula, *R*(*A*/*W*) = *I*_ph_/*PS* where *I*_ph_ is the photocurrent, *P* is the illumination power irradiated onto the channel, *A* is effective area, and *R* is responsivity. The improved photoresponse properties of the Bi_2_S_3_ NW photodetector may be attributed to ultrahigh absorption coefficiency of the Bi_2_S_3_ NW as well as the Schottky contact between the Bi_2_S_3_ NW and Au electrode. The mechanism of charge separation via a Schottky barrier was explained by assisting with a basic energy band theory (Figure S9, Supporting Information). Under light irradiation, photoexcited charges at the Bi_2_S_3_–Au interface are separated quickly due to the existence of the local electric field in the depletion layer. Hence, the electron–hole recombination rate was effectively reduced and the carrier lifetime increased, leading to an increase in free carrier concentration. This process will lower the height of the Schottky barrier and narrow the depletion layer width, thus leading to a significant enhancement in carriers’ tunnelling and transport. Figure 3c,d shows prominent polarization‐dependent photocurrent at bias from 0 to 1 V under the light illumination of near‐infrared (808 nm) and visible light (532 nm). Significantly, it can be seen that the measured photocurrent is highly dependent on the polarization angle and the bias voltage. The photocurrent reached the maximum and minimum values when the incident light polarized along and perpendicular to the *c*‐axis. The photoelectric anisotropy in short‐wave infrared light is more robust than in visible light. Then photocurrents at 1 V with the polarized angle varying from 0 to 360° are extracted and plotted in polar coordinates (Figure 3e,f). The points are fitted by the following function

(9) $$I_{\mathrm{ph}}\left(\theta \right)=I_{\mathrm{pha}}\;\mathrm{co}s^{2}\left(\theta +\varphi \right)+I_{\mathrm{phb}}\mathrm{si}n^{2}\left(\theta +\varphi \right)$$

where *I*_pha_ and *I*_phb_ are photocurrents along *a*‐axis and *b*‐axis of the crystal, respectively, and *θ* is the polarization angle. All fitting results show the gourd shapes and the anisotropy ratio of 1.9 at 808 nm, and 1.41 at 532 nm, respectively. All the optoelectronic properties are summarized in **Table** **1**. With these remarkable features, the low‐dimensional Bi_2_S_3_ photodetctor has the possibility of polarimetric imaging both in near‐infrared region and in visible region.

**Table 1:** Performance of the photodetector based on single Bi_2_S_3_ NW and corresponding dichroisms under the laser with different wavelengths

Wavelength [nm]	Optical power density [mW cm^−2^]	On/off [×10^3^]	Responsivity [A W^−1^]	Dichroic ratio
360	1286.6	0.04	0.012	–
450	9	0.11	6.5	1.31
532	9	10.8	32	1.41
638	9	0.6	8.94	1.13
808	9	0.31	6.82	1.90
1064	207.3	0.003	0.001	1.09

Noise power spectra were measured with a *V*_d_ of 0.5 *V* under short‐wave infrared light (808 nm) with a power density of 11.7 mW cm^−2^ and visible light (532 nm) with a power density of 4.4 mW cm^−2^, respectively. The power spectral density is shown in Figure 3g and Figure S10 (Supporting Information), the photodetector exhibits a lower noise density of 4.39 and 3.94 pA Hz^−1/2^ at the frequency of 1 Hz, respectively. According to the Hooge empirical law: $S_{I}/I_{d}^{\beta }=\alpha_{H}/nf^{\gamma }$, where *S*_I_ is the noise power density, *α*_H_ is the Hooge parameter, *I*_d_ is the current through the detector, *n* is the total number of the carriers, *f* is the frequency, and *β* and *γ* are two fitting parameters. After fitting, *γ* is equal to 0.97, which is very close to 1. Thus, the noise at low frequencies is mainly 1/*f* noise, which is often present in optoelectronic devices due to the fluctuation of transport carriers. The Hooge parameter of various nanomaterials such as Si NWs,^[^ ^38^, ^39^ ^]^ ZnO NWs,^[^ ^40^ ^]^ GaN NWs,^[^ ^41^ ^]^ GaAsSb NWs,^[^ ^42^ ^]^ SnO_2_ NWs,^[^ ^43^ ^]^ InAs NWs,^[^ ^44^ ^]^ CNT,^[^ ^45^ ^]^ bulk silicon,^[^ ^46^ ^]^ SL‐MoS_2_,^[^ ^47^ ^]^ and SL‐graphene^[^ ^48^ ^]^ are compiled in the bar graph of Figure 3h. *α*_H_ (*f* = 1 Hz) of the as‐synthesized Bi_2_S_3_ NWs was estimated to be ≈2 × 10^−5^, considering that *α*_H_ is affected by surface defects, the low value of *α*_H_ observed in the Bi_2_S_3_ NWs can be attributed to the lack of surface defects.^[^ ^49^, ^50^ ^]^ Obviously, the value of *α*_H_ is better than most reported 1D materials‐based devices. The reported performance of Bi_2_S_3_‐based photodetectors synthesized via different approaches and some other nanostructure‐based photodetectors are summarized in **Table** **2**.

**Table 2:** Photodetector performances based on the individual Bi_2_S_3_ NW compared with other nanomaterials

Materials	Wavelength [nm]	Bias [V]	On/off	Rise time	Hooge parameter (*α*_H_)	Reference
Bi_2_S_3_ NSs	632	0.5	20	10 µs	–	^[^ ^35^ ^]^
Bi_2_S_3_ NWs	532	3	15	1 s	–	^[^ ^51^ ^]^
Bi_2_S_3_ NWs	White light	10	1.5 × 10^3^	2.9 s	–	^[^ ^25^ ^]^
Bi_2_S_3_ NWs	532	2	1.08 × 10^4^	20 ms	1.6 × 10^−5^	This work
Bi_2_S_3_ NWs	808	2	1.3 × 10^3^	20 ms	2.0 × 10^−5^	This work
InAs NWs	532	15	10^2^	–	1.4 × 10^−1^	^[^ ^44^, ^52^ ^]^
ZnO NWs	370	5	–	0.1 ms	4 × 10^−2^	^[^ ^40^, ^53^ ^]^
GaN NWs	360	5	10^3^	–	2 × 10^−2^	^[^ ^41^, ^54^ ^]^
GaAsSb NWs	1330	0.15	–	–	2.2 × 10^−3^	^[^ ^42^, ^55^ ^]^
SnO_2_ NWs	275	5	2.99 × 10^5^	60 ms	4 × 10^−2^	^[^ ^43^, ^56^ ^]^
Si NWs	638	5	–	–	10^−6^–10^−3^	^[^ ^38^, ^39^, ^57^ ^]^
Bulk silicon	–	–	–	–	10^−5^–10^0^	^[^ ^46^ ^]^
CNT	Infrared	1	1.1 × 10^4^	–	9.3 × 10^−3^	^[^ ^45^, ^58^ ^]^
SL‐MoS_2_	561	8	10^6^	4	5.7 × 10^−3^	^[^ ^47^, ^59^ ^]^
SL‐graphene	1550	0.4	–	–	4 × 10^−4^	^[^ ^48^, ^60^ ^]^

According to McWhorter's model,^[^ ^48^ ^]^ carrier capture and emission back to the channel leads to the current fluctuations *δI∝q(δn)µ*, *μ* is the mobility and *q* is the charge of an electron. The power spectral density is described as following, $\frac{S_{I_{d}}}{I_{d}^{2}}={\left(\frac{g_{m}}{I_{d}}\right)}^{2}\;\frac{e^{2}k_{B}T\lambda N}{WLfC_{i}^{2}}$ where *I*_d_, *g*_m_, *k*_B_, *λ*, *N*, *f*, and *C*_i_ are the drain current, transconductance, Boltzmann constant, tunnel attenuation length, trap density of states, frequency, and capacitance of gate dielectric, respectively. Electron–phonon coupling and vacancy capture electron are the main reasons for the mobility fluctuation. Defects act as carrier traps play an important role in tailoring the optoelectronic properties of materials and adjusting 1/*f* noise, which is consistent with the previous report.^[^ ^28,49,50^ ^]^ In this work, the sulfur‐assisted vapor transport preparation method reduces S vacancies of the Bi_2_S_3_ NWs, increasing optoelectronic performances while reducing 1/*f* noise. The combination of superior photoresponse properties and lower electrical noise in Bi_2_S_3_ NWs provides excellent material and device platform for polarized imaging application.

**Figure 4:**
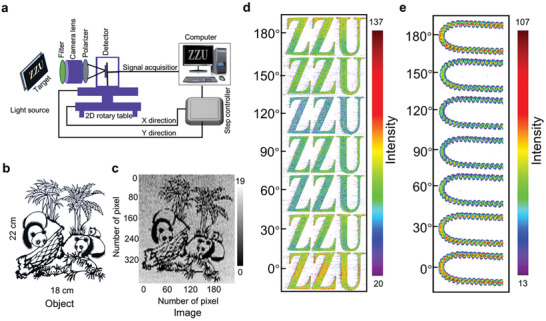
Polarization imaging based on Bi_2_S_3_ NW photodetector. a) A schematic diagram of polarization imaging measurement system. b) The object of the paper‐cut of panda (18 × 22 cm^2^). c) Visible (532 nm) light polarization image (with 216 × 404 pixels) using the 2D imaging system. d,e) 2D color maps of the abbreviation of Zhengzhou University (ZZU) and U‐shaped helical tube under 532 and 808 nm with polarization angle from 0° to 180°, respectively.

Due to the excellent polarization photoelectric performance of the device, direct polarization imaging realized by constructing a single Bi_2_S_3_ NW image sensor (Figure S11, Supporting Information). **Figure** **4**a describes the schematic illustration of the polarization imaging measurement system. The imaging targets was a paper‐cut (Figure 4b) or “ZZU” (Figure S13a, Supporting Information) or self‐luminous U‐shaped helical tube (Figure S14a, Supporting Information). The image sensor was equipped with a dark camera box on a 2D rotary table. The imaging targets were captured by the camera lens and focused on the image sensor. The Bi_2_S_3_ image sensor was configured as a point‐like detector to collect the light, which is recorded by a computer. To acquire a whole image information, the image sensor scanned the target line by line by controlling the movements of the 2D rotary table (Figure S12, Supporting Information). Images with a resolution of 216 × 404 pixels for paper‐cut and 210 × 121 pixels for a U‐shaped tube are successfully obtained, as shown in Figure 4c (Figures S13b, S14b, and S15, Supporting Information). Especially for the paper‐cut target, the details of bamboo leaf can be well tracked and expressed, exhibiting a good contrast of imaging. The results suggest that the image sensor based on 1D Bi_2_S_3_ NW have dual‐band imaging capability. In this work, we summarized with different rotation angle 2D color map of “ZZU” letter under 532 nm with different rotation angles (Figure 4d) and “U‐shaped helical tube” under 808 nm (Figure 4e) with the polarization angle (*θ*) of incident light change from 0° to 180°. The Bi_2_S_3_ NW‐based image sensor indicated the two‐band angle‐dependent polarized imaging capability. To further improve the efficiency of polarization imaging, the polarization imaging system constructed with Bi_2_S_3_ NWs photodetectors array is considered in future study.

## 3 Conclusion

In summary, 1D Bi_2_S_3_ NWs with high crystallinity were successfully synthesized via a sulfur‐assisted vapor transport growth method. Strong anisotropy of 1D layered Bi_2_S_3_ has been demonstrated experimentally and theoretically. This work provides a strategy to increase optoelectronic performances and reduce 1/*f* noise via a facile sulfurization approach. Significantly, the Bi_2_S_3_ NW based device shows a superior device performance in terms of high photoresponsivity, broadband, high dichroic ratio, and excellent polarization imaging. Importantly, the noise for the device is much lower than other low‐dimensionanl semiconductors and comparable to the ultralow noise of silicon nanowires. Direct polarimetric imaging has been implemented at the wavelengths of both 532 nm (visible) and 808 nm (near‐infrared) without the help of any normal used optical polarizer. Our work paves a potential pathway toward constructing low noise dual‐band polarimetric image sensor based on 1D materials.

## 4 Experimental Section

### Growth of Bi_2_S_3_ Nanowires

The Bi_2_S_3_ nanowires were synthesized via a sulfur‐assisted vapor transport. Specifically, sulfur powder (Aladdin, 99.99%) and bismuth sulfide powder (Aladdin, 99.99%) were used as the S and Bi precursors. The alumina boat with ≈10 mg bismuth sulfide powder was placed at the center of the quartz tube. The alumina boat containing ≈30 mg sulfur powders was located upstream with a distance of ≈9.5 cm from the tube center. A piece of SiO_2_/Si substrate (10 mm × 10 mm in size) was placed downstream side with a distance of ≈8.5 cm from the center. The furnace temperature of the central zone was ramped to growth temperature (670 °C) within 30 min and maintained for 8 min for the growth of Bi_2_S_3_ crystals. 35 sccm Ar gas was used as carrier gas in the reaction process. The furnace was naturally cooled down to room temperature with flowing Ar gas. As a result, a certain amount of large‐size Bi_2_S_3_ nanowires were synthesized on the Si/SiO_2_ substrates.

### Characterization of Bi_2_S_3_ NWs

The crystal structure of Bi_2_S_3_ NWs was characterized by X‐ray diffraction using Bruker D8 ADVANCE. Raman microscope and spectrometer was collected by Renishaw in Via Reflex with an excitation laser of 785 nm. The Bi_2_S_3_ NWs were transferred onto carbon film‐supported copper grids and observed on TEM. The single‐crystalline nanowire was confirmed in TEM collected by JEOL2100F. The morphology and diameter of Bi_2_S_3_ NWs were further confirmed by optical microscopy and atomic force microscopy (AFM) (Bruker Dimension Icon). Angle‐resolved Raman spectra of Bi_2_S_3_ were measured by microscopic confocal laser Raman spectrometer (InVia, Renishaw, excited by the 532 nm laser). The noise power spectra were measured by a PDA (Platform Design Automation, Inc.).

### Device Fabrication and Measurements

The two‐terminal photodetection devices based on the Bi_2_S_3_ nanowires were fabricated on the SiO_2_/Si substrate. Au electrodes (45 nm) were evaporated by thermal evaporation. The photoelectric properties of Bi_2_S_3_ NWs device were carried out in ambient conditions by using a probe station equipped with a semiconductor device analyzer (B1500A Keysight). Laser with different wavelength including 360, 450, 532, 638, 808, and 1064 nm were used as a light source. Polarization‐dependent photoresponse was measured by rotating the polarization of the incident light using a half‐wave plate in the light path under the 532 and 808 nm laser diodes, respectively. The noise power spectra were measured by a PDA (Platform Design Automation, Inc.).

### DFT Calculations

The structures, electronic, and optical properties of Bi_2_S_3_ were simulated via using the VASP^[^ ^33^, ^34^ ^]^ based on the DFT by performing the generalized gradient approximation (GGA) and projected augmented wave (PAW) to modulate the exchange‐correlation functional and electron–ion potential, respectively. The kinetic energy cutoff of 450 eV and the Gamma k‐mesh of 15 × 15 × 1 as parameters were used to calculate the band structure of Bi_2_S_3_ nanowires. 20 Å thickness of vacuum region was used to simulate the nanowire structures. The convergence criteria of energy and forces were chosen as 10^−5 ^eV and 0.01 eV Å^−1^, respectively. HSE06 methods were also used to calculate the electronic band structures and optical absorption of Bi_2_S_3_ nanowire accurately.

## Conflict of Interest

The authors declare no conflict of interest.

## Supporting information

Supporting Information

Supplemental Movie 1

Supplemental Movie 2

## Data Availability

Research data are not shared.
