# Supplementary material for: Low‐Noise Dual‐Band Polarimetric Image Sensor Based on 1D Bi2S3 Nanowire
Source: Adv Sci (Weinh). 2021 May 21;8(14):2100075. doi: 10.1002/advs.202100075 (PMC8292854; doi:10.1002/advs.202100075)
Supplement: Supplementary file 1 — Supporting Information [file ADVS-8-2100075-s002.pdf]

WILEY-VCH

© 2021 Wiley-VCH GmbH

ADVANCED SCIENCE
Open Access

Supporting Information

for *Adv. Sci.,* DOI: 10.1002/advs.202100075

Low-Noise Dual-band Polarimetric Image Sensor based on 1D Bi_2S_3 Nanowire

Wen Yang, Juehan Yang, Kai Zhao, Qiang Gao, Liyuan Liu, Ziqi Zhou, Shijun Hou, Xiaoting Wang, Guozhen Shen, Xinchang Pang, Qun Xu, Zhongming Wei**

Supporting Information

Low-Noise Dual-band Polarimetric Image Sensor based on 1D Bi_2S_3 Nanowire

Wen Yang, Juehan Yang, Kai Zhao, Qiang Gao, Liyuan Liu, Ziqi Zhou, Shijun Hou, Xiaoting Wang, Guozhen Shen, Xinchang Pang, Qun Xu, Zhongming Wei**

W. Yang, Prof. X. Pang, Prof. Q. Xu
School of Materials Science and Engineering, Zhengzhou University, Zhengzhou 450052, China
E-mail: xuqun@zzu.edu.cn

W. Yang, Dr. J. Yang, K. Zhao, Q. Gao, Prof. L. Liu, Z. Zhou, S. Hou, Dr. X. Wang, Prof. G. Shen, Prof. Z. Wei
State Key Laboratory of Superlattices and Microstructures, Institute of Semiconductors, Chinese Academy of Sciences, Beijing 100083, China
E-mail: zmwei@semi.ac.cn

K. Zhao, Prof. L. Liu, Z. Zhou, S. Hou, Prof. G. Shen, Prof. Z. Wei
Center of Materials Science and Optoelectronics Engineering, University of Chinese Academy of Sciences, Beijing 100049, China

Prof. Q. Xu
Henan Institute of Advanced Technology, Zhengzhou University, Zhengzhou 450052, China

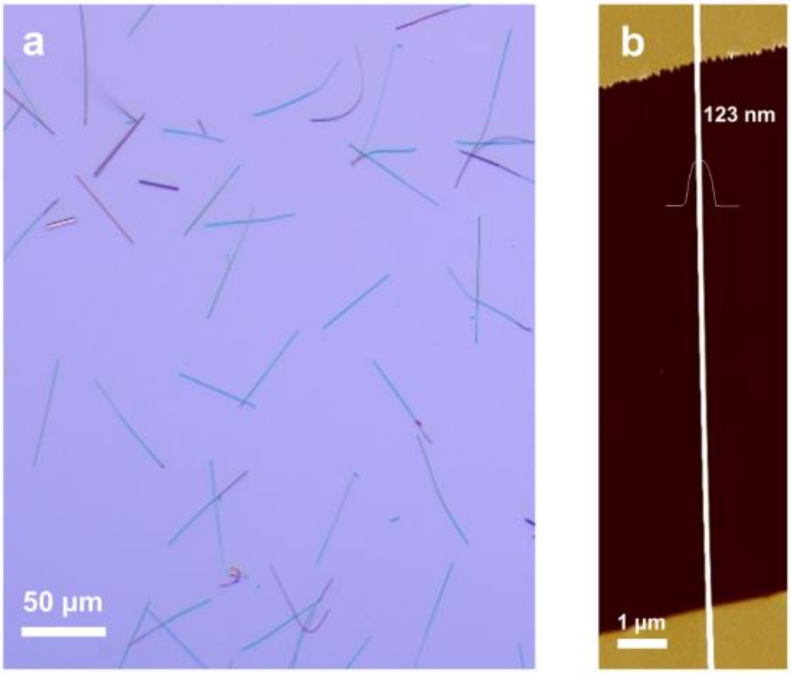

Figure S1. a) Typical microscopy optical image of synthesized 1D Bi_2S_3 Nanowires on SiO_2/Si substrate. b) AFM topography of Bi_2S_3 photodetector with diameter of 123 nm.

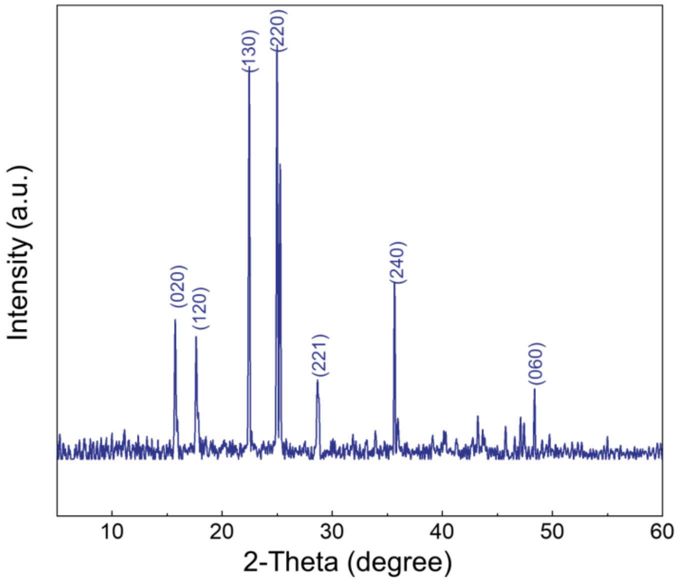

Figure S2. XRD pattern of Bi_2S_3 Nanowires

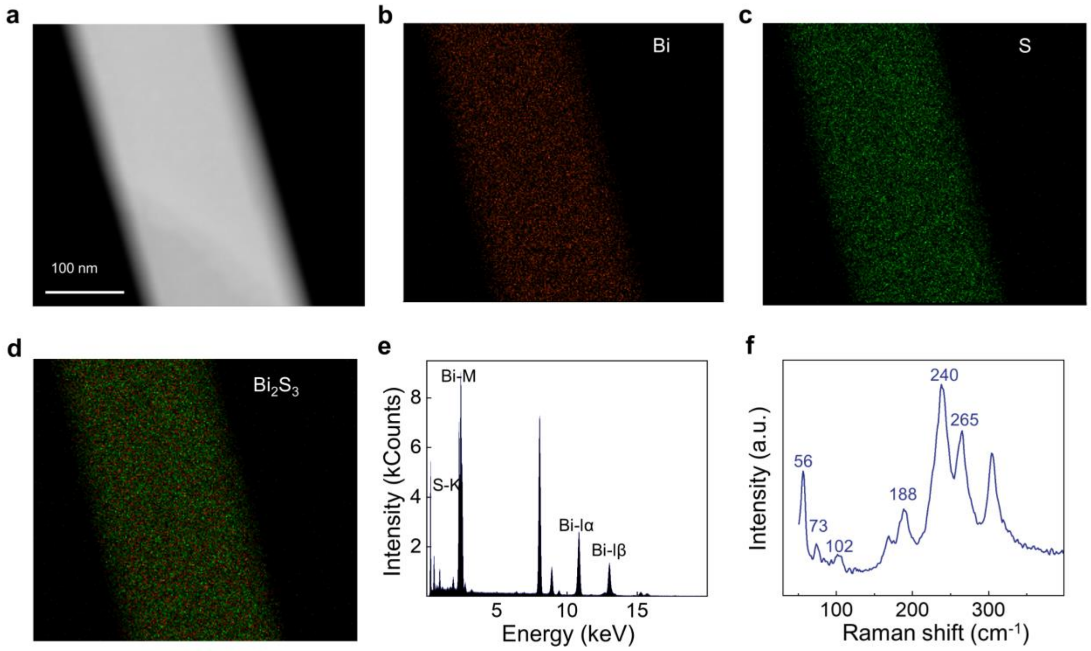

Figure S3. a-d) HAADF image c) of the Bi_2S_3 NW and corresponding elemental maps for b) S, c) Bi and d) mixture of S and Bi. e) TEM-EDX of the Bi_2S_3 nanowire. f) Raman spectrum of Bi_2S_3 nanowire.

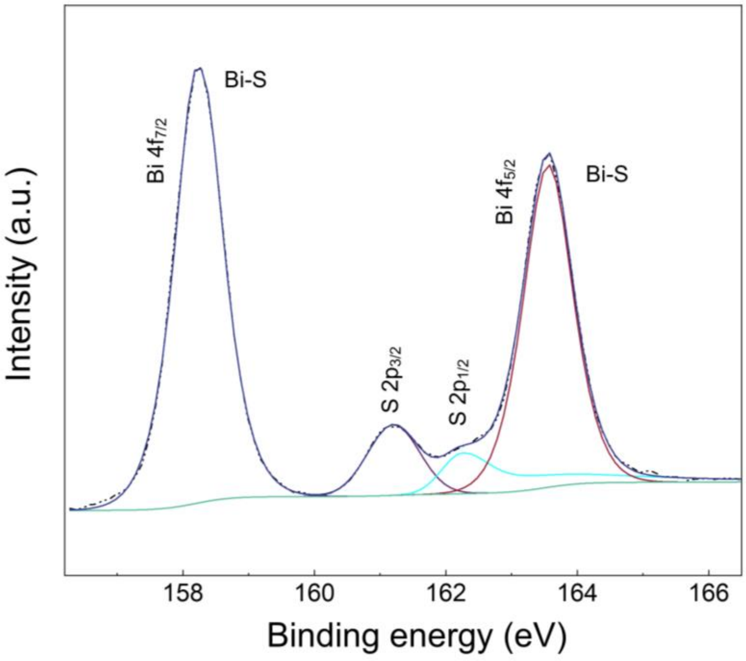

Figure S4. High-resolution XPS spectrum of Bi_2S_3 nanowires.

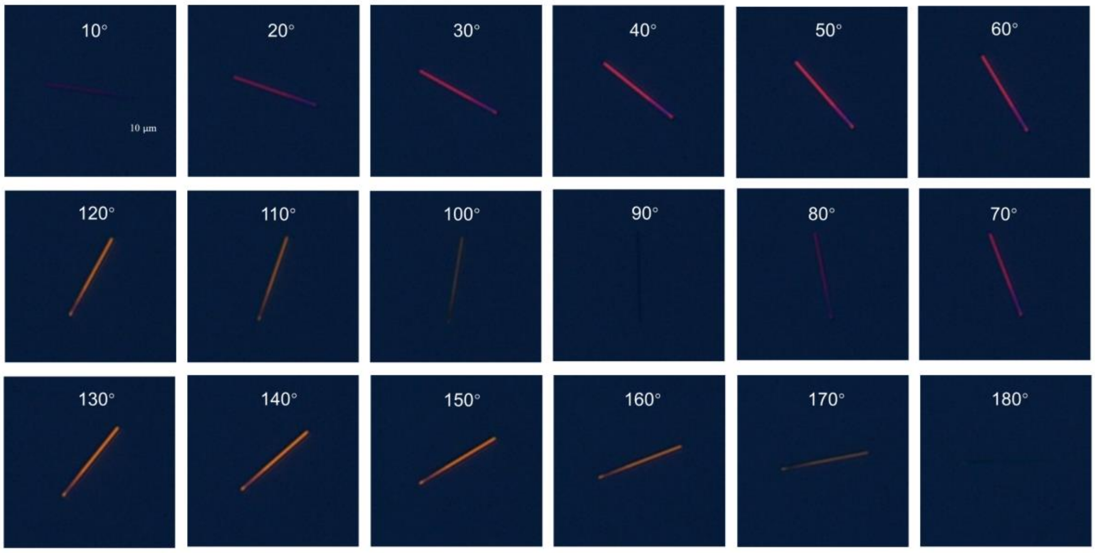

Figure S5. PROM images of the Bi_2S_3 Nanowire on SiO_2/Si substrate under cross-polarized light illumination from 0° to 180°.

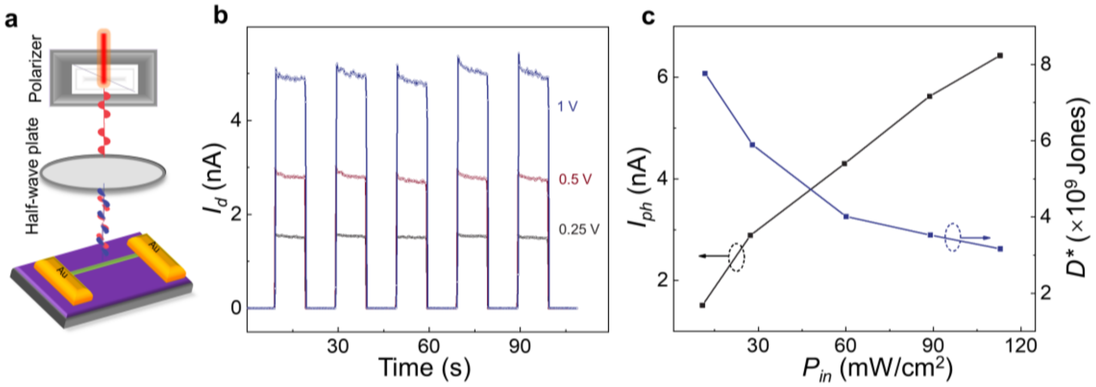

Figure S6. a) 3D Schematic diagram of the photodetection device based on Bi_2S_3. b) Drain current I_d as a function of time with light switched on/off at V_d = 0.25, 0.5, and 1 V. c) photocurrent and detectivity (D^*) of the Bi_2S_3 photodetector at various light intensities. Note: the wavelength of laser is 808 nm.

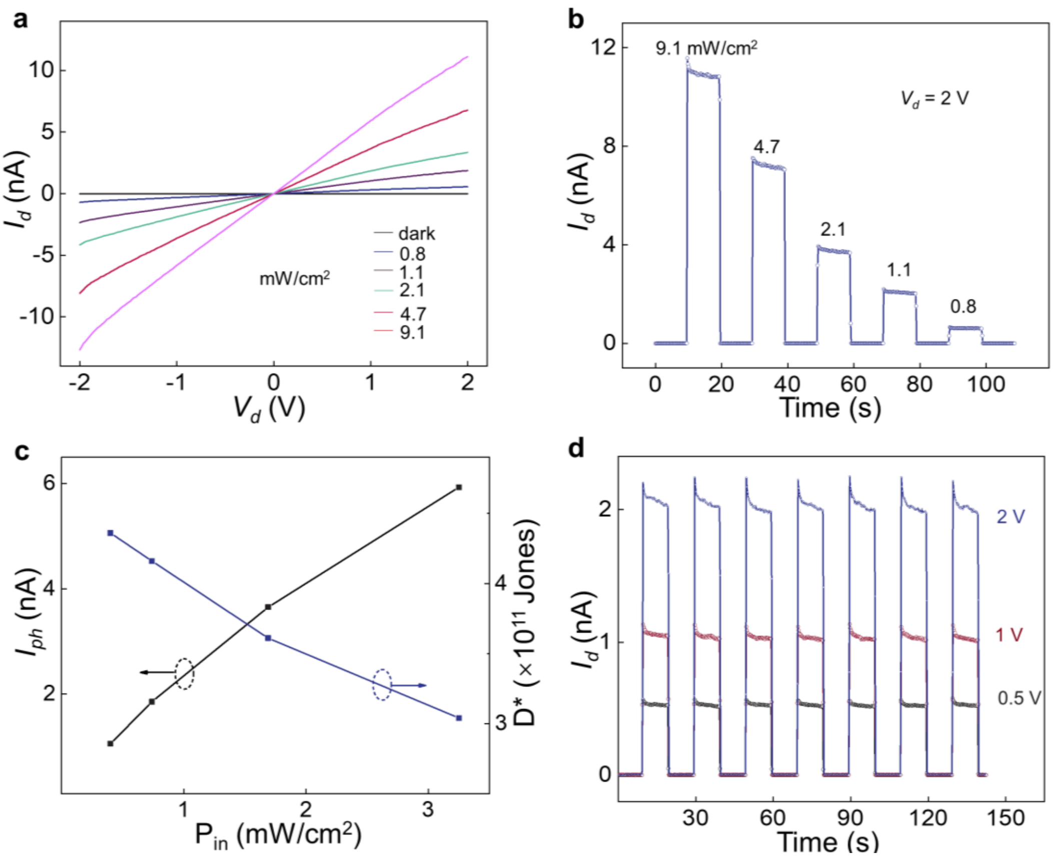

Figure S7. a) I_d-V_d plots of Bi_2S_3 photodetector under 532 nm light illumination at different power densities. b) Photoresponse of the device irradiated under different power density from 0.08 to 9.1 mW/cm^2. c) Photocurrent and detectivity (D^*) of the Bi_2S_3 photodetector at various light intensities. d) Drain current I_d as a function of time with light switched on/off at V_d = 0.5, 1, and 2 V. Note: the wavelength of laser is 532 nm.

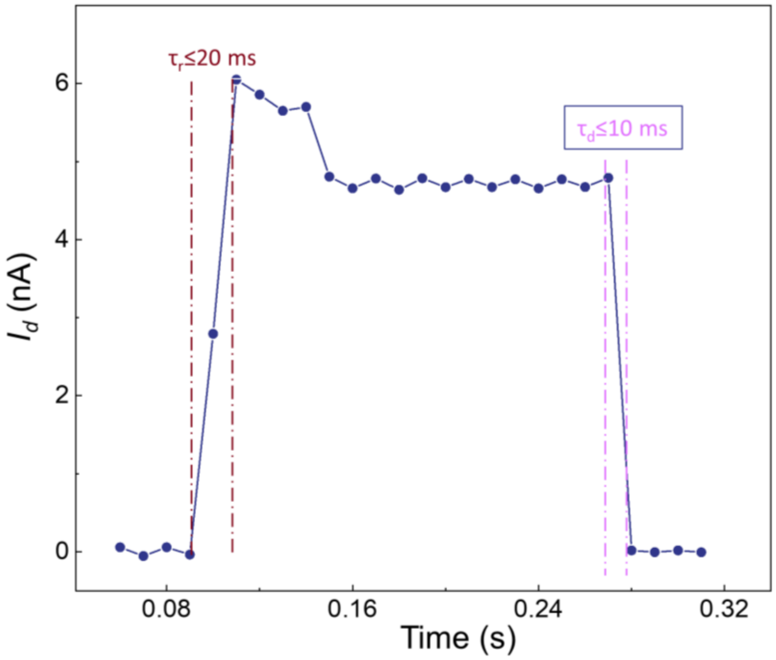

Figure S8. Time-resolved photoresponse of the Bi_2S_3 device with rise and decay time of 20 and 10 ms, respectively.

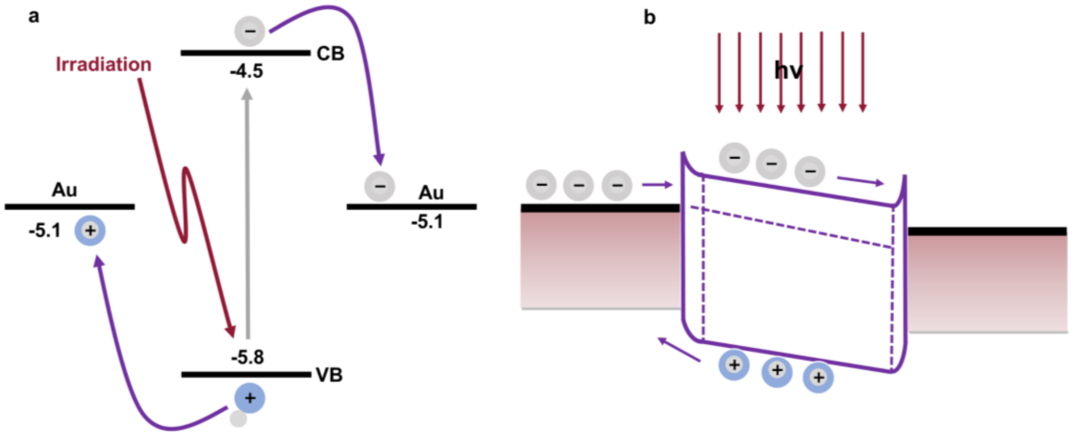

Figure S9. a) Energy level diagrams of the Bi_2S_3 NWs and Au films. b). Band diagrams of the Bi_2S_3 NW photodetector under irradiation condition.

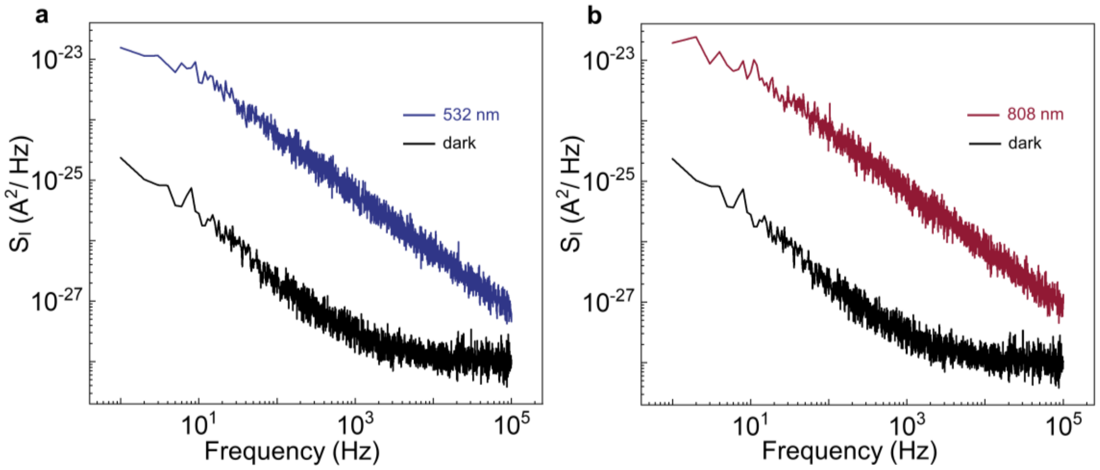

Figure S10. Low-frequency noise measurement on the device.

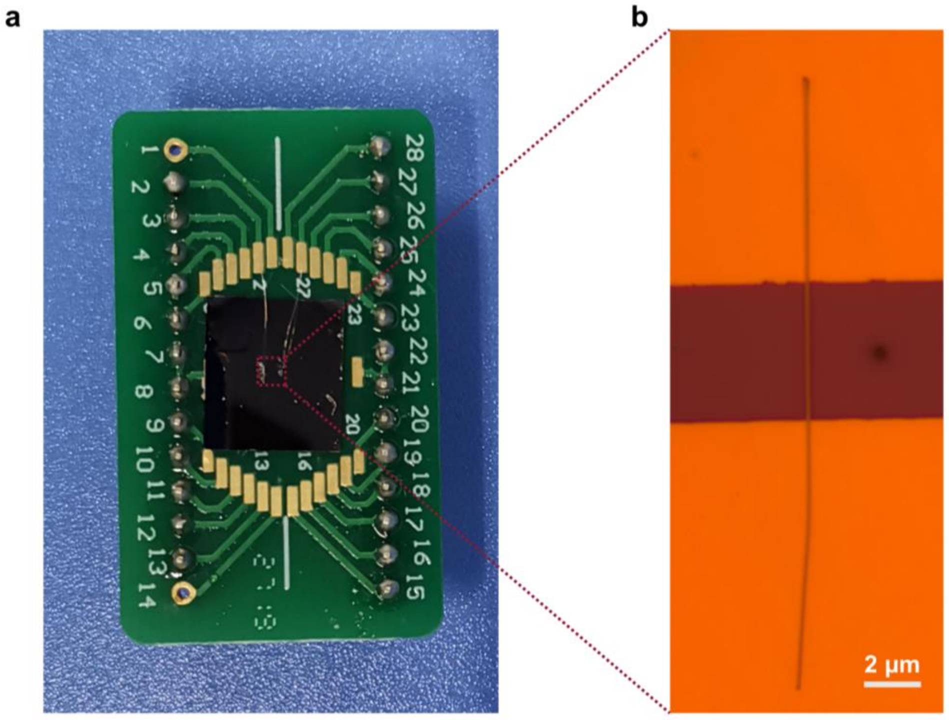

Figure S11. a) Digital photograph of the image sensor. b). Microscope image of the single Bi_2S_3 nanowire sensor used for polarization imaging.

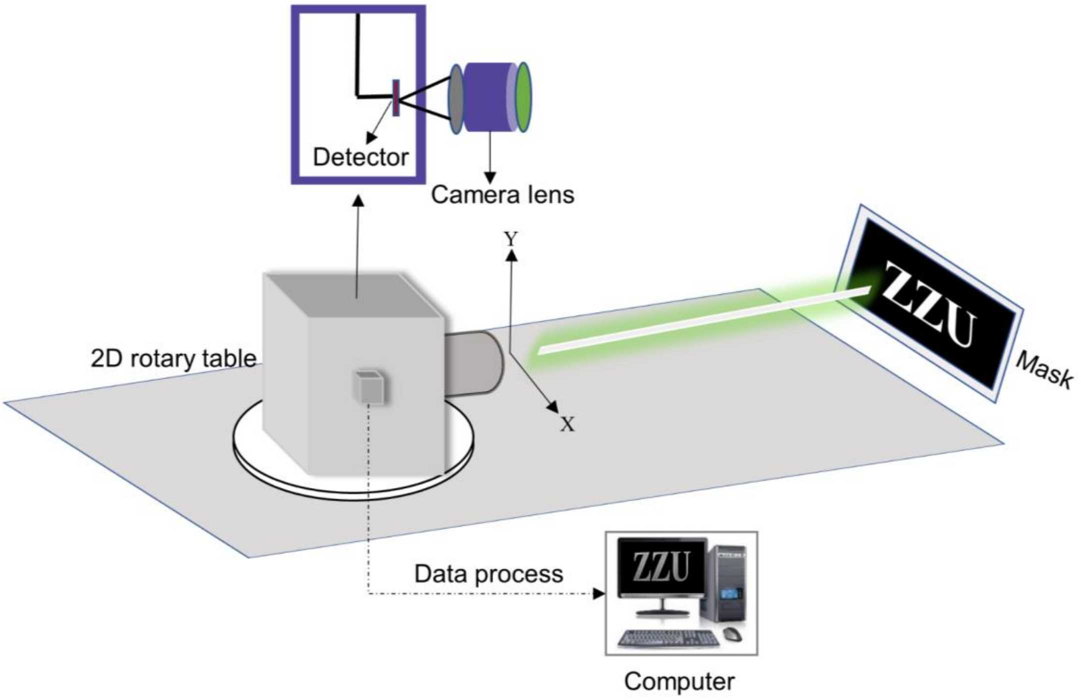

Figure S12. Schematic diagram of imaging measurement system.

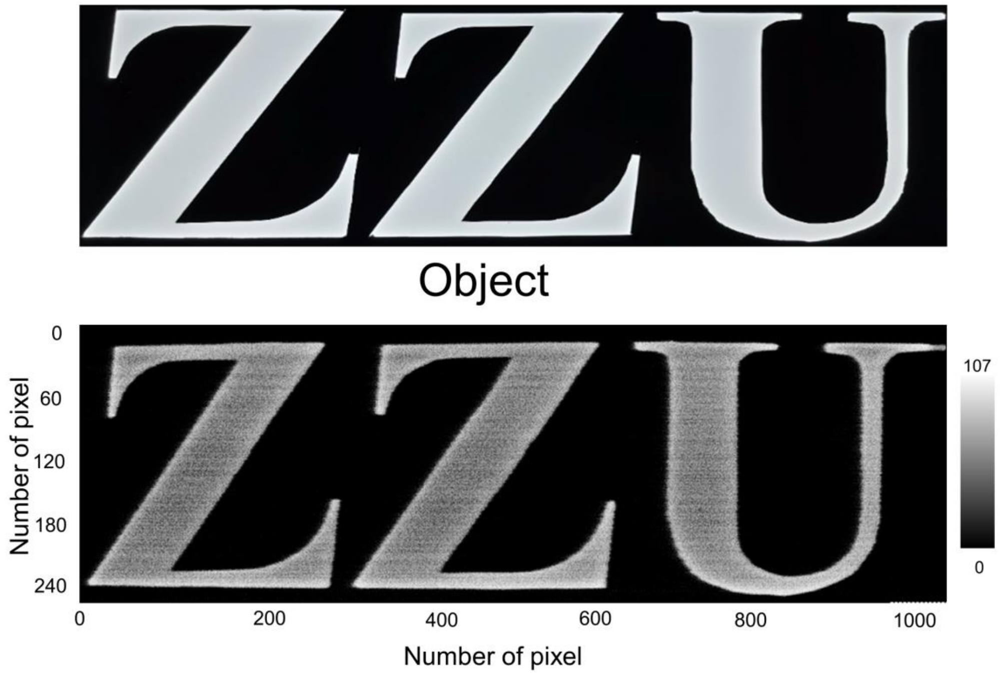

Figure S13. Visible (532 nm) light polarization image (with 261×1020 pixels) using the two-dimensional imaging system.

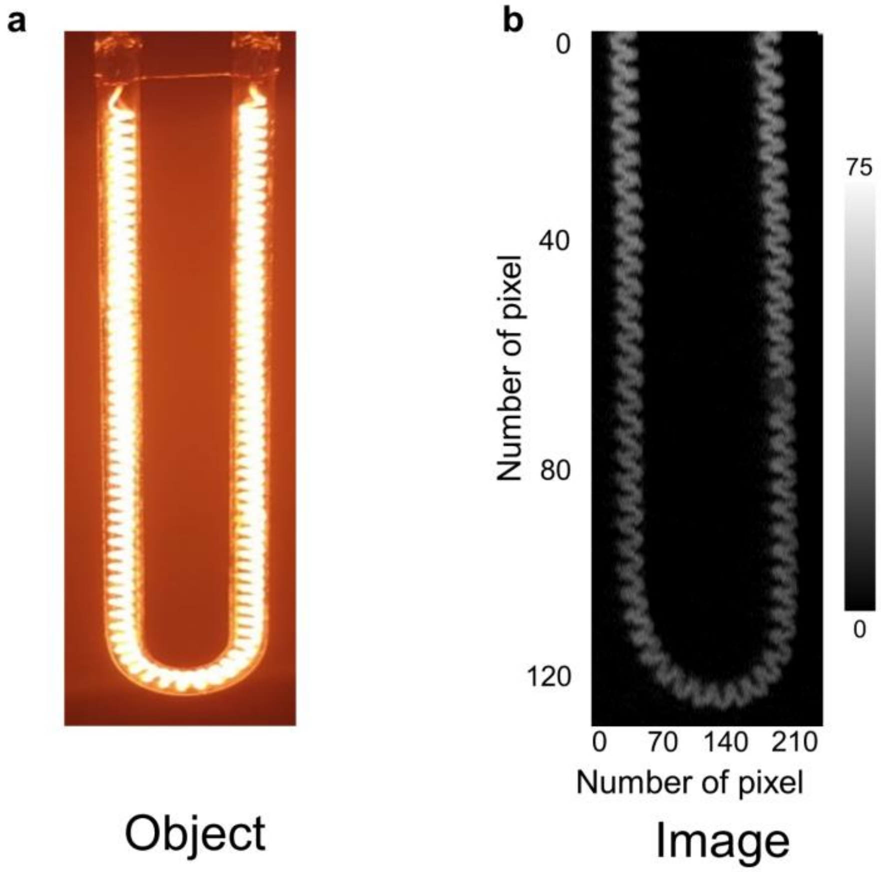

Figure S14. a) Digital image of U-shaped helical tube. b) Near-infrared (808nm) light polarization image (with 210×121 pixels) using the two-dimensional imaging system.

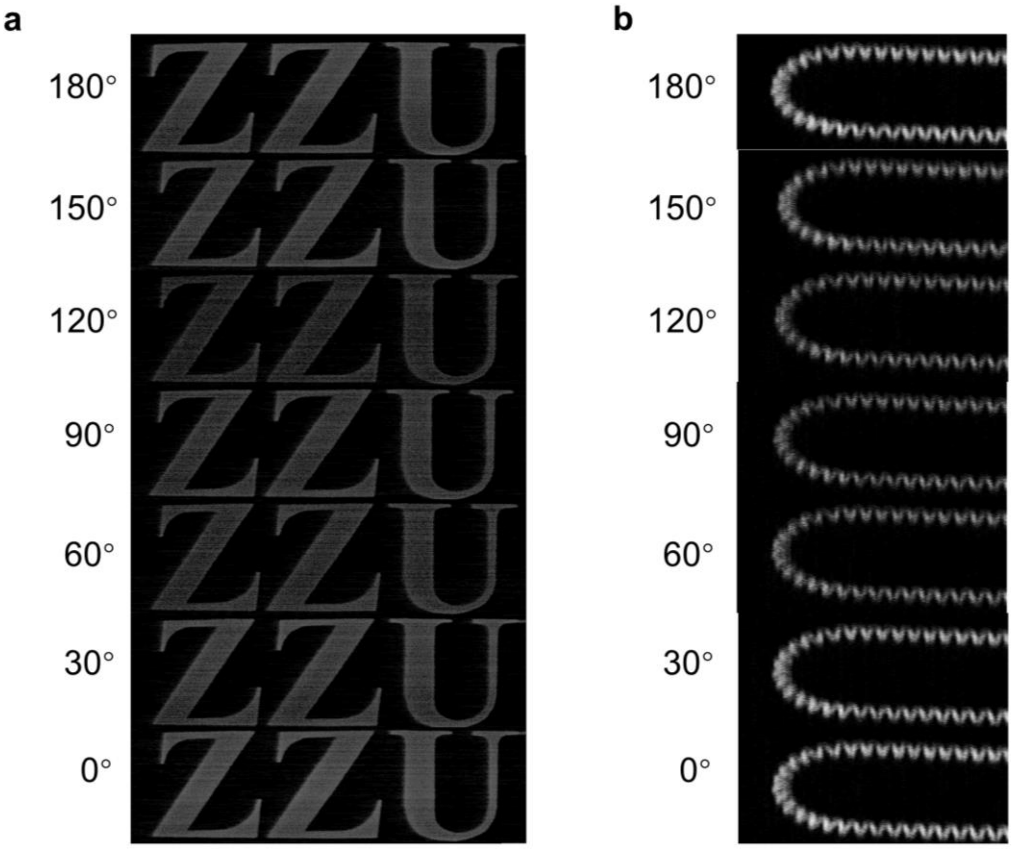

Figure S15. a) Visible (532 nm) and b) Near-infrared (808nm) light polarization image using the two-dimensional imaging system with polarization angle from 0° to 180°, respectively.
